# The effect of surgical repair of hiatal hernia (HH) on pulmonary function: a systematic review and meta-analysis

**DOI:** 10.1007/s10029-023-02756-5

**Published:** 2023-02-24

**Authors:** Y. Wang, Y. Lv, Y. Liu, C. Xie

**Affiliations:** 1 General Surgery Department, Shanxi Bethune Hospital, Shanxi Academy of Medical Sciences, Tongji Shanxi Hospital, Third Hospital of Shanxi Medical University, Taiyuan, 030032 China; 2 Tongji Hospital, Tongji Medical College, Huazhong University of Science and Technology, Wuhan, 430030 China

**Keywords:** Hiatal hernia, Paraoesophageal hernia, Surgical repair, Operation, Pulmonary function

## Abstract

**Purpose:**

Hiatal hernia is renowned for the symptom of reflux, and few physicians associate a hiatal hernia with pulmonary issues. It is widely acknowledged that a hiatal hernia can be treated with surgery. However, less is known about how the surgical procedure would benefit pulmonary function. Thus, the aim of this study was to determine whether surgical repair can improve pulmonary function in patients with hiatal hernias.

**Methods:**

We registered the protocol on the PROSPERO (International Prospective Register of Systematic Reviews) platform (no. CRD42022369949). We searched the PubMed, Embase, Cochrane Library, and ClinicalTrials.gov databases for cohort studies that reported on the pulmonary function of patients with hiatal hernias. The quality of each cohort study was evaluated using the Newcastle–Ottawa scale (NOS). We then calculated mean differences (MDs) with 95% confidence intervals for these continuous outcomes. Each study’s consistency was appraised using the *I*^*2*^ statistic. The sensitivity analysis was performed using the trim-and-fill method. Publication bias was confirmed using the funnel plot visually and Egger regression test statistically.

**Results:**

A total of 262 patients from 5 cohorts were included in the meta-analysis. The quality evaluation revealed that, of these 5 papers, 3 received 8 NOS stars out of 9 stars, 1 received 9, and the other received 7, meaning all included cohort studies were of high quality. The results showed that surgical repair for a hiatal hernia significantly improved forced expiratory volume in 1 s (FEV1; weighted mean difference [WMD]:0.200; 95% CI 0.047–0.353; *I*^2^ = 71.6%; *P* = 0.010), forced vital capacity (FVC; WMD: 0.242; 95% CI 0.161–0.323; *I*^2^ = 7.1%; *P* = 0.000), and total lung capacity (TLC; WMD: 0.223; 95% CI 0.098–0.348; *I*^2^ = 0.0%; *P* = 0.000) but had little effect on residual volume (RV; WMD: –0.028; 95% CI –0.096 to 0.039; *I*^2^ = 8.7%; *P* = 0.411) and the diffusing capacity carbon monoxide (DLCO; WMD: 0.234; 95% CI –0.486 to 0.953; *I*^2^ = 0.0%; *P* = 0.524).

**Conclusion:**

For individuals with hiatal hernias, surgical repair is an efficient technique to improve respiratory function as measured by FEV1, FVC, and TLC.

**Supplementary Information:**

The online version contains supplementary material available at 10.1007/s10029-023-02756-5.

## Introduction

Hiatal hernia is a condition in which abdominal viscera protrude through the esophageal hiatus of the diaphragm and into the mediastinum of the chest [1]. Several factors contribute to the development of the disease, including obesity and aging [2]. Depending on how far and deep the hernia invasion has progressed, there are four subtypes of hiatal hernias: types I through IV [3]. The gastroesophageal junction slides above the diaphragm in type I hernias. In type II, the fundus protrudes while the gastroesophageal junction remains in situ. The herniation of the fundus and gastroesophageal junction into the chest is classified as type III. The presence of a structure in addition to the stomach in the hernia sac, such as the spleen, colon, or intestine, distinguishes type IV hiatal hernias from other types. Despite the fact that type I hiatal hernias account for more than 95% of cases and most are asymptomatic, some conditions, such as strangulation of the hernia contents and gastric volvulus, can be life threatening. The related symptoms include digestive symptoms, such as regurgitation, dysphagia, dyspepsia, or reflux, and extra respiratory symptoms, such as dyspnea, cough, hoarseness, or anemia [3]. For all symptomatic hiatal hernias, surgical repair is necessary. Therefore, perceiving symptoms and ascertaining what role the hernias play in those symptoms is of crucial importance. However, the majority of clinical manifestations appear to be nonspecific, making it hard to determine the true extent of hiatal hernia impact. Despite difficulties in identifying surgical indications, several studies have demonstrated that surgical repair of hiatal hernias alleviates symptoms such as dysphagia, heartburn, chest pain, and reflux and is well received by the majority of patients with only minimal morbidity and mortality [4–7]. Improvement of pulmonary symptoms following hiatal hernia surgery is also supported by several subjective studies, which strengthen the widespread belief that surgical repair improves respiratory function in patients treated for hiatal hernias [8–11]. Nevertheless, the pulmonary issues in these patients have long been overlooked. In our experience, the majority of physicians do not associate chronic respiratory issues with hiatal hernias. Furthermore, some researchers have delved deeply into the subject and offered valuable data about respiratory function in recent years [5, 12–17]. Therefore, the purpose of this systematic review and meta-analysis is to assess the effect of surgical repair of hiatal hernias on pulmonary function in terms of quantitative results. In doing so, we aim to assist medical professions in in-clinic patient counseling and improve decision-making for patients.

## Methods

We followed the guidelines of PRISMA (Preferred Reporting Items for Systematic Reviews and Meta-Analyses) to conduct this meta-analysis [18]. The protocol was preregistered in PROSPERO (International Prospective Register of Systematic Reviews; approval no. CRD42022369949).

### Data sources and search strategy

We searched the PubMed, the Cochrane Library, Embase, Web of Science, and Clinictrial.gov for cohort studies released from the inception of the database to September 27, 2022. The search method combined the use of keywords and medical topic headings (MeSH), and there were no language constraints. The search terms used were “paraoesophageal hernia,” “hiatal hernia,” and “pulmonary” or “symptom improvement” or “dyspnea” or “outcomes” or “quality of life” and “surgery” individually or in combination. The search strategy for this review is presented in the supplementary material (Table 1–4). The search was expanded using the “related articles” feature, and the relevance of each reference from included studies was taken into account.

### Eligibility criteria and study selection

Studies were included if they reported on type I–IV hiatal hernia, were cohort studies, reported on patients who received any surgical treatment, and provided pre-operative and post-operative respiratory function parameters. Studies were excluded if they reported on patients who had previously undergone gastric operations and/or provided only subjective records of respiratory symptoms. Based on these predefined inclusion and exclusion criteria, the research selection was carried out separately by two authors (Changhu Xie and Yanhua Liu). Any disagreements were settled through discussion between these two authors.

### Data extraction

Two authors (Yanhua Liu and Changhu Xie) independently extracted data from eligible trials and recorded the data into a previously designed Microsoft Excel sheet. If any discrepancies emerged, the authors would discuss them to arrive at a consensus. The following information was extracted from each study: author, year, country, patient number, age, gender, BMI, type of hiatal hernia, open surgical procedure, laparoscopic surgical procedure, and type of fundoplication. The primary outcome was forced expiratory volume in one second (FEV1), and the secondary outcomes were forced vital capacity (FVC), residual volume (RV), total lung capacity (TLC), and the diffusing capacity for carbon monoxide (DLCO). Forced expiratory volume in one second (FEV1) is known as the highest amount of air that an individual may forcibly evacuate in the first second after maximal inhaling [19]. The entire amount of air expelled during the FEV test is known as forced vital capacity (FVC) [20]. Residual volume (RV) is the amount of air still present in the lungs after maximally vigorous expiration. In other words, it is the volume of air that the lungs are unable to exhale, thus leaving the alveoli permanently open [21]. Carbon monoxide diffusing capacity (DLCO) is also known as transfer factor for carbon monoxide, and is also an indicator of the conductivity of gas from inspired gas to red blood cells [22].

### Quality assessment

The quality of each cohort study was evaluated by the Newcastle–Ottawa scale (NOS) [23]. The number of stars given to a study, with 0 being the lowest quality and 9 being the highest quality, signifies the quality of the study. For each cohort study, 4 stars were awarded for participant selection and exposure measurement, 2 stars for comparability, and 3 stars for outcome assessment and adequate follow-up. Scores of 0–3, 4–6, and 7–9 were regarded as corresponding to low, moderate, and excellent quality, respectively.

### Statistical analysis

All statistical analyses were performed using Stata Statistical Software version 14.0 (StataCorp, College Station, TX, USA). We calculated mean differences (MDs) with 95% CIs for continuous outcomes, which were FEV1, FVC, RV, TLC, and DLCO. The *I*^*2*^ statistic, a quantitative outcome of study inconsistency, was used to assess heterogeneity across studies. Studies with an *I*^*2*^ statistic between 25 and 50%, between 50 and 75%, and over 75% were regarded as having low, moderate, and high heterogeneity, respectively. If the *I*^*2*^ value was greater than 50%, meta-analyses were carried out using a random-effects model; otherwise, a fixed model was adopted. The sensitivity analysis was performed by omitting one study each time and then re-running the meta-analysis procedure to verify the robustness of the overall effects. Visual inspection of the funnel plot was used to confirm publication bias, and Egger regression test was applied to determine publication bias statistically.

## Results

### Trial selection

**Fig. 1:**
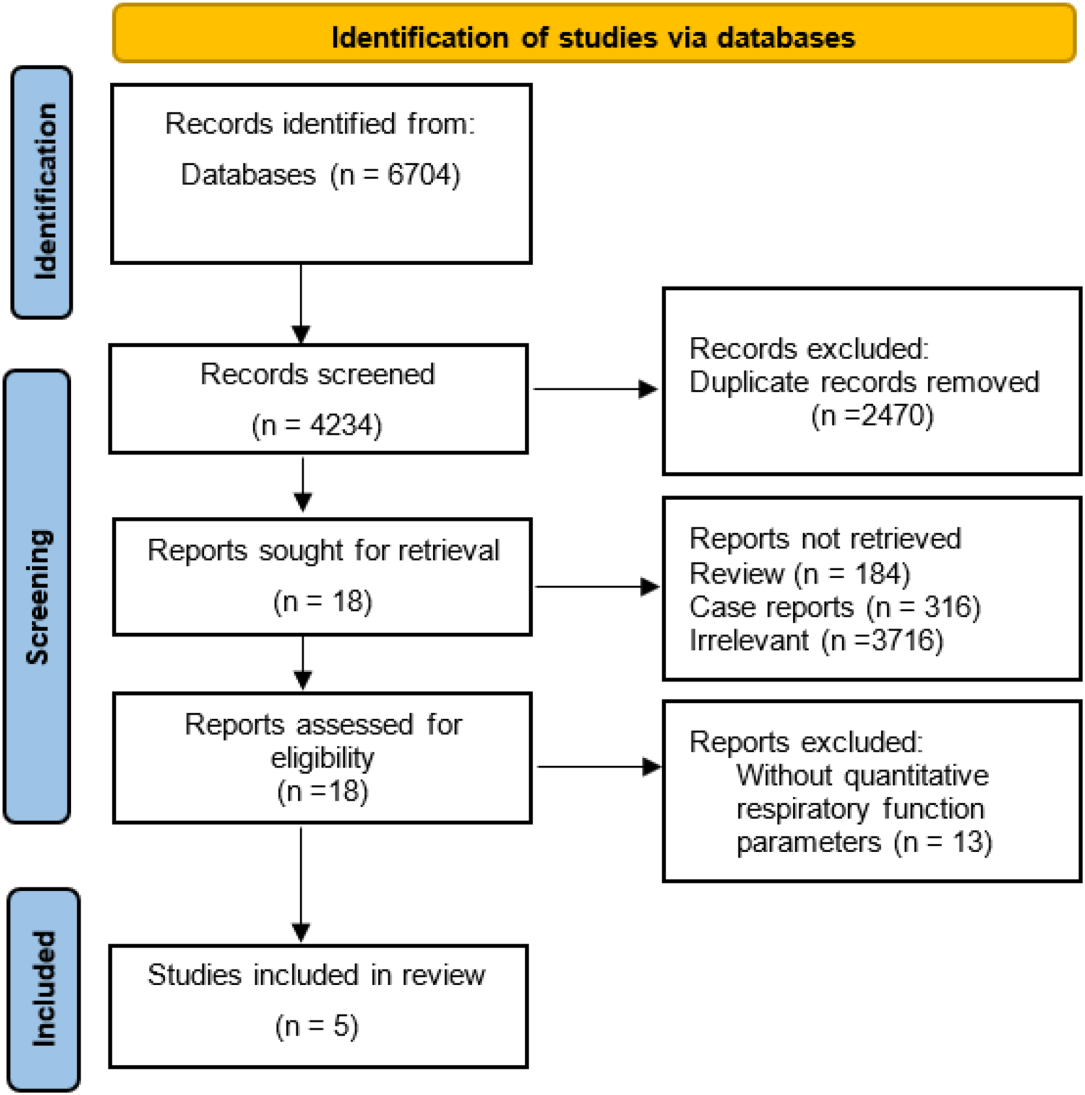
PRISMA flow diagram of included cohort studies

We performed a comprehensive search, and a total of 6704 articles were found in electronic databases. After 683 duplicates were removed, 6021 articles were screened for their titles and abstracts. Then 184 reviews, 316 case reports, 1787 conference abstracts, and 3734 irrelevant articles were eliminated. There remained 18 articles to be identified for full-text review. Of these, 13 articles were excluded because none provided qualified quantitative respiratory function measures. Finally, 5 cohorts were included [13–17]. The selection process for the included cohort studies in the meta-analysis is shown in Fig. 1.

### Study characteristics

All included trials were published between 2002 and 2022. The sample size of each individual trial ranged from 30 to 73. The majority of participants were 60 years of age or older. The majority of participants were female. All five studies only recruited patients with hiatal hernia of types II to IV except for Naoum et al. [15], whose study published in 2017 included one patient of type I hiatal hernia. Regarding surgery, four of the five eligible studies opted for the laparoscopic method, while the study by Low and Simchuk [17], published in 2002, included participants who underwent open abdominal procedure. The characteristics of the eligible cohort studies are presented in Table 1**.**

**Table 1:** Basic characteristics of included studies

Author	Year	Country	Study type	Study duration	Patients (*n*)	Age	Male/female	Type of HH	Surgical procedure (Open), *n*	Surgical procedure (LAP), *n*	Surgical approach
Aiolfi [14]	2022	Italy	Cohort	2015–2020	71	Median (IQR) 67.1 (13.1)	15/56	II–IV	0	71	Toupet
Bouriez [13]	2022	France	Cohort	2019–2021	43	Median (range) 70 (63.0–73.6)	6/37	II–IV	0	43	Toupet
Low [17]	2002	USA	Cohort	1999–2001	45	Mean (range) 71.5 (46, 91)	16/29	II–IV	45	0	Hill
Naoum [15]	2017	Australia	Cohort	2009–2012	73	Mean (SD) 70 (10)	20/54	I–IV	0	73	NR
Naoum [16]	2011	Australia	Cohort	2009–2010	30	Mean (SD) 70 (10)	7/23	III–IV	2	28	Nissen + Gastropexy

*LAP* laparoscopy, *HH* hiatal hernia

### Quality assessment

We assessed the quality of each included article according to the criteria of the NOS for cohort studies. All cohort studies included in this meta-analysis were of good quality, as indicated by scores of 7 or higher for each trial. The scores of the eligible studies are shown in Table 2.

**Table 2:** Newcastle–Ottawa quality assessment scale for cohort studies

Author	Year	Selection	Comparability	Outcome	NOS
Aiolfi [14]	2022	★★★★	★	★★	8
Bouriez [13]	2022	★★★	★★	★★★	8
Low [17]	2002	★★★★	★★	★★	8
Naoum [15]	2017	★★★	★★	★★	7
Naoum [16]	2011	★★★★	★★	★★★	9

### Primary outcome

#### Meta-analysis of FEV1

**Fig. 2:**
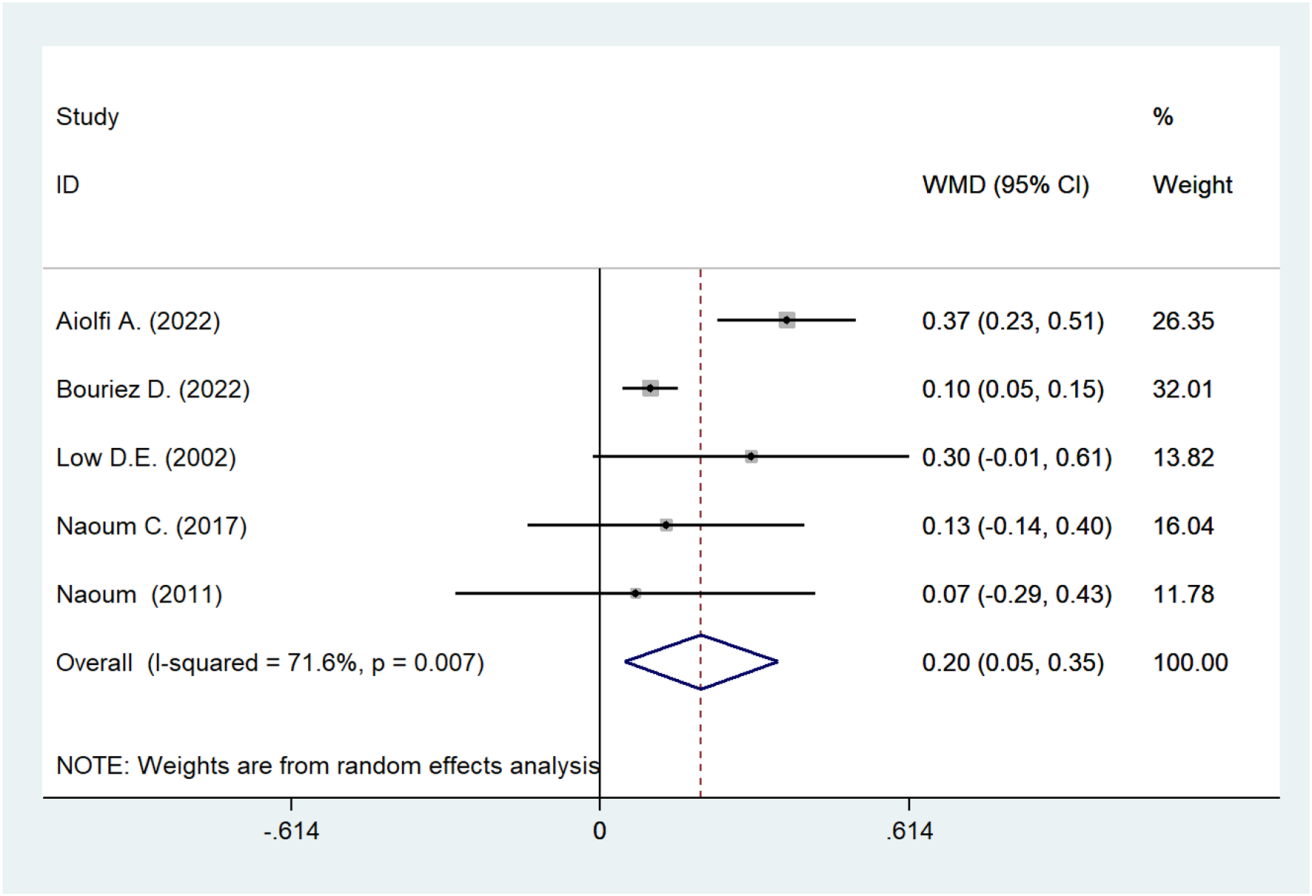
Meta-analysis of post-operative FEV1. *FEV1* forced expiratory volume in 1 s

The forest plot showed that the post-operative FEV1 increased significantly compared to pre-operative FEV1 yet with heterogeneity. We chose the random-effects model to perform the final meta-analysis, and the final combined value was 0.2, which meant that hiatal hernia repair surgery improved the FEV1 (weighted mean difference [WMD]: 0.200; 95% CI 0.047–0.353; *I*^2^ = 71.6%; *P* = 0.010; Fig. 2). The level of between-study heterogeneity was moderate. Therefore, we conducted a sensitivity analysis. The results showed that the exclusion of any single study did not bias the result, which corroborated the robustness of this meta-analysis (See Supplementary Fig. 1).

### Secondary outcomes

#### Meta-analysis of FVC

**Fig. 3:**
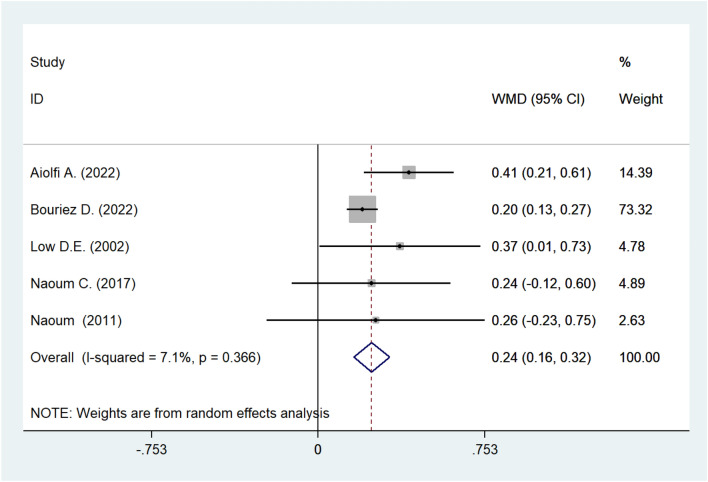
Meta-analysis of post-operative FVC. *FVC* forced vital capacity

Five cohort studies explored the association between the change in FVC and the surgical repair for hiatal hernia. The pooling analysis showed that, after surgery, FVC increased significantly (WMD: 0.242; 95% CI: 0.161–0.323; *I*^2^ = 7.1%; *P* < 0.001; Fig. 3).

#### Meta-analysis of TLC

**Fig. 4:**
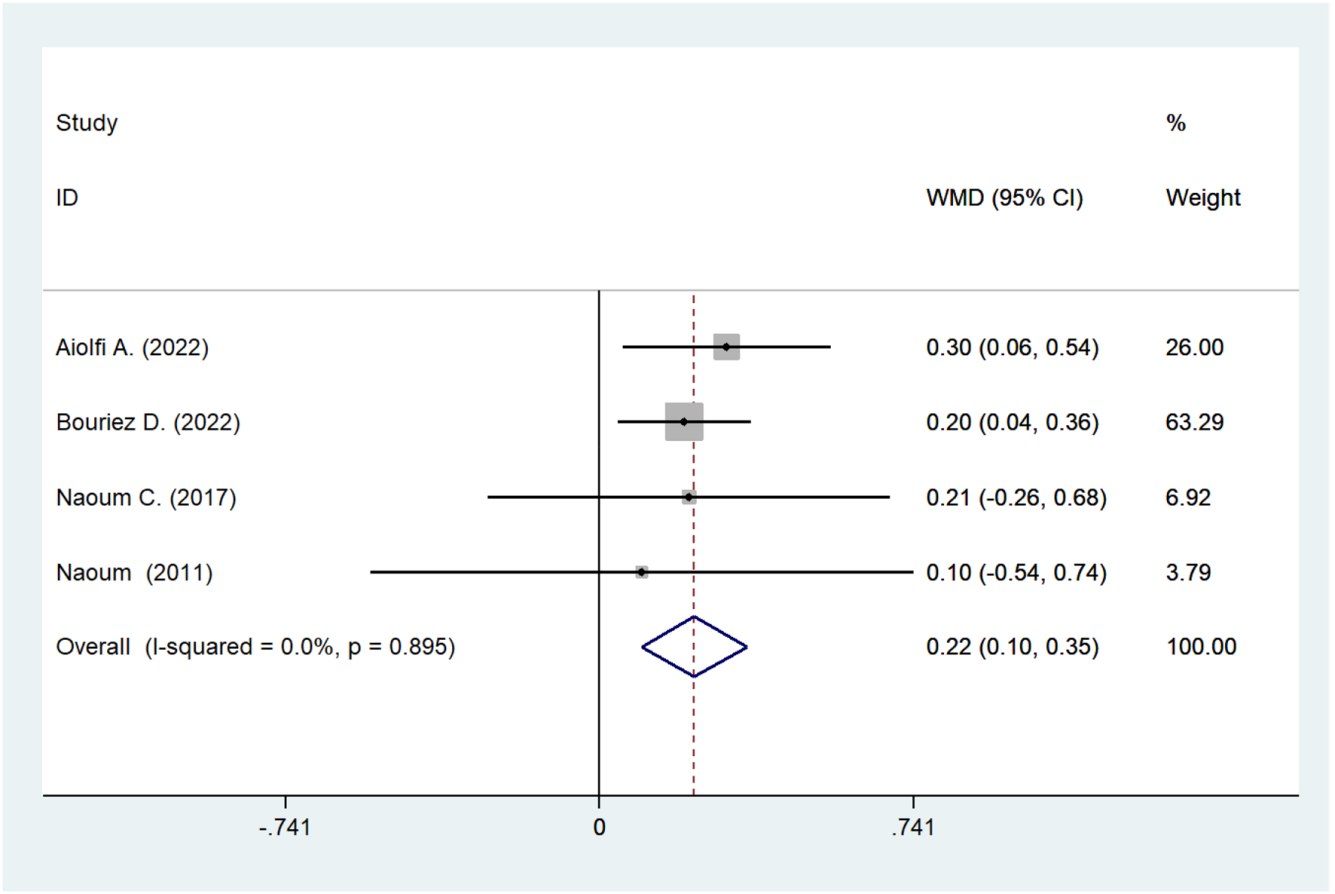
Meta-analysis of post-operative TLC. *TLC* total lung capacity

The pre- and post-operative numerical value of TLC was documented in four cohort studies. The synthesized statistic indicated that surgery ameliorated TLC (WMD: 0.223; 95% CI 0.098–0.348; *I*^2^ = 0.0%; *P* = 0.000; Fig. 4).

#### Meta-analysis of RV

**Fig. 5:**
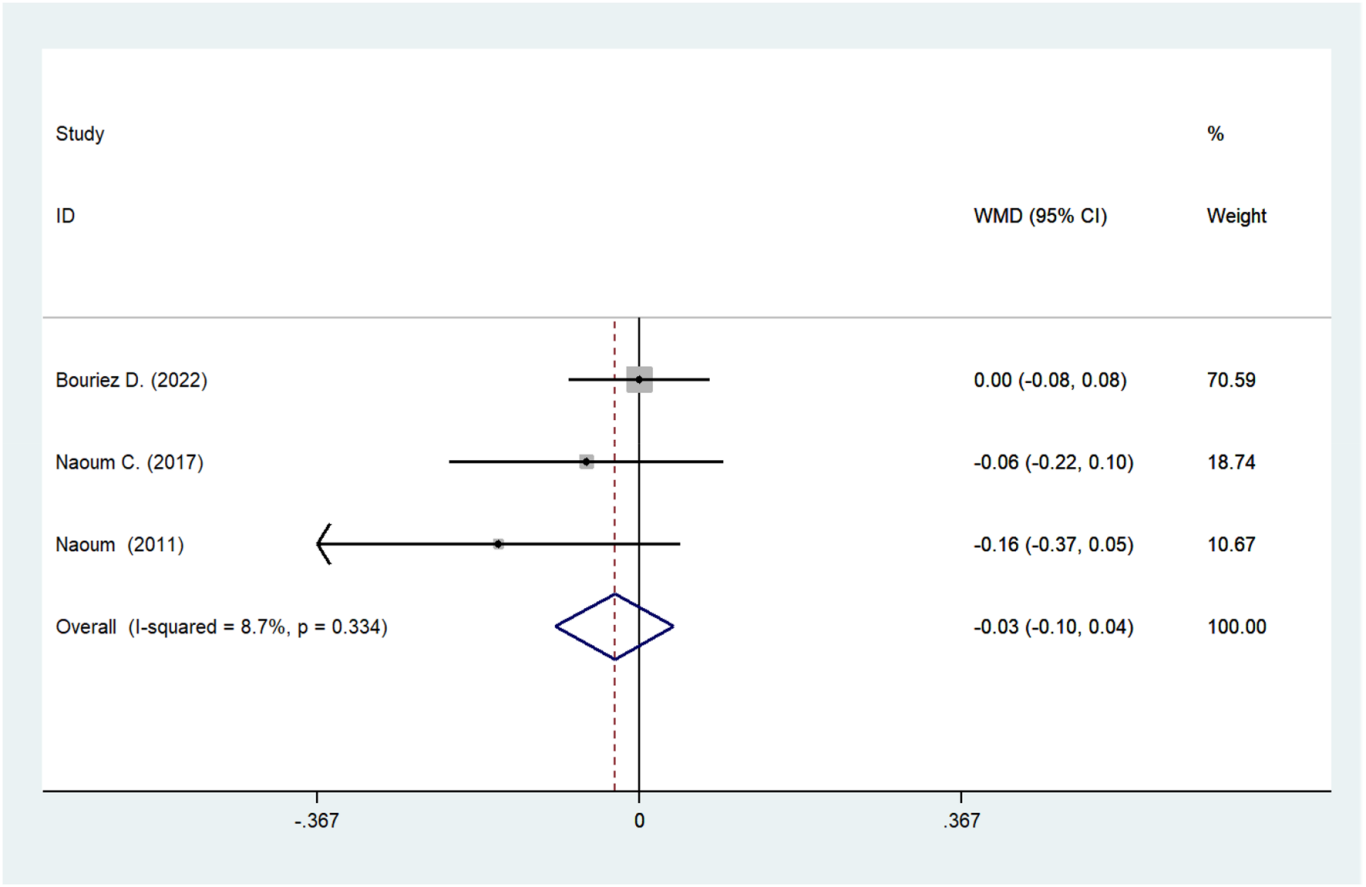
Meta-analysis of post-operative RV. *RV* residual volume

The records of RV before and after surgery were only available in three studies. The combined consequence signified that post-operative RV seemed lower than that of pre-operative RV but without statistical significance (WMD: –0.028; 95% CI: –0.096 to 0.039; *I*^2^ = 8.7%; *P* = 0.411; Fig. 5).

#### Meta-analysis of DLCO

**Fig. 6:**
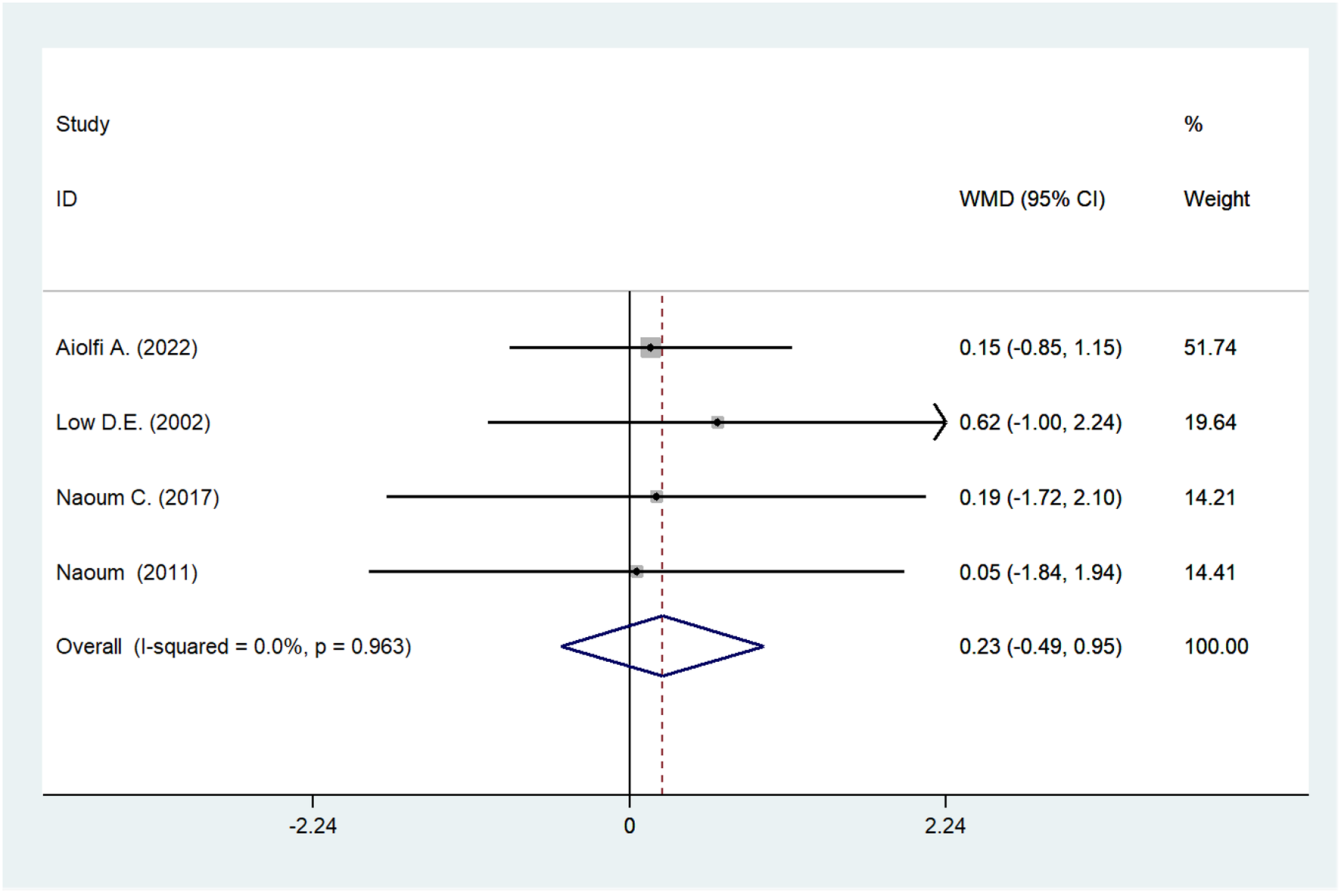
Meta-analysis of post-operative DLCO. *DLCO* diffusing capacity carbon monoxide

Four cohort studies documented the DLCO before and after surgery. Our meta-analysis showed that repair surgery for hiatal hernia improved DLCO, but without significance (WMD: 0.234; 95% CI –0.486 to 0.953; *I*^2^ = 0.0%; *P* = 0.524; Fig. 6).

#### Publication bias

**Fig. 7:**
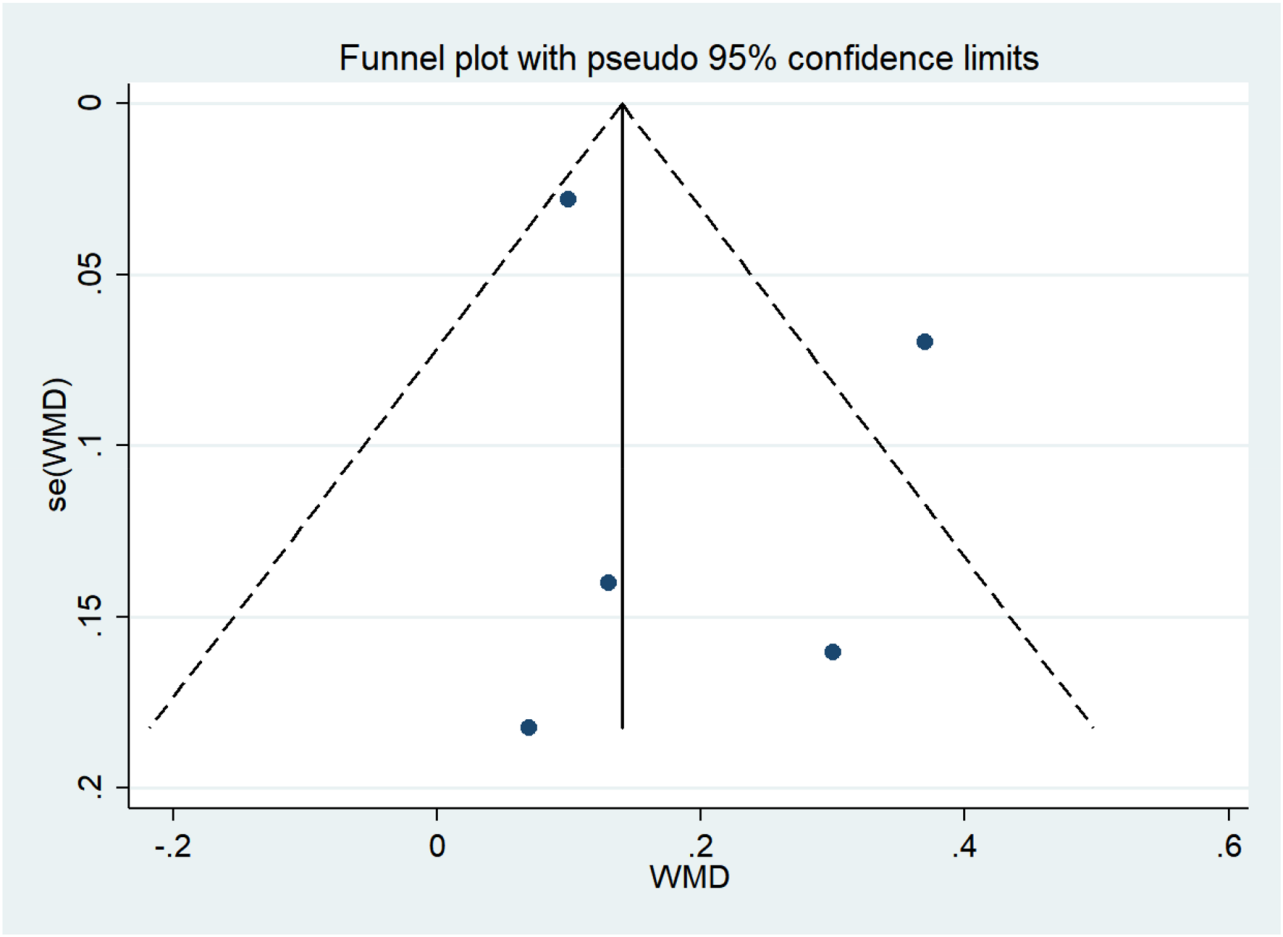
Publication bias of the risk of FEV1

A visual examination of the funnel plot revealed no signs of major publication bias in the FEV1 results (Fig. 7). Additionally, Egger’s regression test (*P* = 0.446 > 0.05) revealed no publication bias in our meta-analysis (See Supplementary Fig. 2).

## Discussion

This is the first study to examine the relationship between pulmonary function and the repair of hiatal hernias through meta-analysis and systematic review. We incorporated 5 cohort studies totaling 262 participants, offering the most thorough analysis of the relationship between pulmonary function and hiatal hernia surgical repair. According to our research, FEV1, FVC, and TLC showed statistically significant improvement after surgical repair, whereas RV and DLCO showed nonsignificant improvement. These results suggest that surgical correction may be beneficial for patients experiencing pulmonary discomfort with hiatal hernia.

Upper gastrointestinal symptoms, such as regurgitation, dysphagia, retention, and reflux, have traditionally been the main focus of evaluating the clinical consequences of hiatal hernias. The majority of medical professionals do not associate having hiatal hernias with chronic respiratory issues. However, prior studies revealed that the prevalence of pre-operative respiratory symptoms ranged from 30 to 44%, indicating that the pulmonary issues in hiatal hernia patients have been disregarded (8, 16). Patients with hiatal hernias are frequently older aged and often have additional concomitant conditions that could cause respiratory problems. Despite these confounding elements, some trials demonstrated that surgical correction significantly improved pulmonary function [5, 24–26]. However, there has not yet been a study that examines how surgical repair affects pulmonary function.

Theoretically, an improvement in the FEV1 and FVC indicates the release of airway obstructions brought on by the constriction of the respiratory outlets by the abdominal viscera and the reduction of restrictions brought on by the increased volume available for lung expansion. Sometimes, unsatisfactory performance in FEV1 and FVC is attributed to poor expel of the patients; therefore, the post-operative increase may be representative of systemic improvements, such as exercise capacity, food intake, and cardiac function [6], in addition to better respiratory function. Improvements in TLC and FVC were postulated to be the result of the physical removal of abdominal organs or tissue from the thoracic cavity [27]. A lack of RV changes (WMD: –0.028; 95% CI: –0.096 to 0.039; *I*^2^ = 8.7%; *P* = 0.411) after the operation in these eligible investigations further support this observation. Our results suggest that the presence of intrathoracic abdominal viscera may decrease pulmonary functional capacity. This finding is further reinforced by the fact that patients who underwent HH repair experienced significant increases in TLC and FVC after surgery. The current meta-analysis revealed that post-operative DLCO varied insignificantly, which means that the source of lung issues among patients with hiatal hernia has little association with the pathological changes of pulmonary tissue, which is much common in conditions like pulmonary fibrosis, pulmonary edema, and silicosis. This further substantiates the hypothesis that pulmonary compression is the mechanism underlying pulmonary problems due to hiatal hernia.

Even though all preoperational measures of pulmonary function were within the normal range, and the slight improvement after the surgery might have been statistically nonsignificant, this does not imply that it was perceptibly negligible. Another study found that 53% of people who did not report dyspnea before surgery experienced an improvement in their breathing [8]. Although this study only examined subjective sensation, our research provided additional quantitative support for its findings.

The present study is unique because it is the first to quantitatively evaluate the impact of the surgical correction on pulmonary function and because registration and reporting were standardized. However, there were still some limitations. First, all the included studies were retrospective, and prospective trials are needed. Second, the equipment was a significant source of variation between and within laboratories. Equipment from different manufacturers has demonstrated accuracy differences of as much as 20% over a 3-month period [28]. None of the articles we included offered any information about the testers, which might have compromised the results. Third, only five cohort studies were included in this review, without subgroup analysis of hiatal hernia size and type. Future studies could further explore the effects of hiatal hernia repair and pulmonary function around the size and type of hiatal hernia. Finally, we concluded that FEV1, FVC, and TLC were improved significantly after operation. However, it is unclear whether statistical significance equates to clinical significance, and thus further research is warranted.

## Conclusion

Surgical correction of hiatal hernia improved FEV1, FVC, and TLC significantly but did little to RV and DLCO. These results serve as a reminder to healthcare professionals during in-clinic patient counseling that respiratory issues, in addition to digestive symptoms, may be caused by hiatal hernias, and that surgical repair can objectively improve the patient’s pulmonary function.

## Supplementary Information

Below is the link to the electronic supplementary material.

Supplementary file1 (DOCX 49 KB)
