# Supplementary material for: The effect of surgical repair of hiatal hernia (HH) on pulmonary function: a systematic review and meta-analysis
Source: Hernia. 2023 Feb 24;27(4):839–48. doi: 10.1007/s10029-023-02756-5 (PMC10374806; doi:10.1007/s10029-023-02756-5)

**The retrieval strategies and retrieval results of each database are shown in Tables**

**Table 1** Pubmed

| No. | Content | result |
| --- | --- | --- |
| #1 | Search: "Hernia, Hiatal"[Mesh] Sort by: Most Recent | [6,707](https://pubmed.ncbi.nlm.nih.gov/?sort=date&term=%22Hernia%2C+Hiatal%22%5BMesh%5D) |
| #2 | Search: ((((((((((((((((((((((((((((((((Hernias, Hiatal[Title/Abstract]) OR (Hiatal Hernias[Title/Abstract])) OR (Hernia, Hiatus[Title/Abstract])) OR (Hernias, Hiatus[Title/Abstract])) OR (Hiatus Hernia[Title/Abstract])) OR (Hiatus Hernias[Title/Abstract])) OR (Hiatal Hernia[Title/Abstract])) OR (Sliding Hiatal Hernia[Title/Abstract])) OR (Hernia, Sliding Hiatal[Title/Abstract])) OR (Hernias, Sliding Hiatal[Title/Abstract])) OR (Hiatal Hernia, Sliding[Title/Abstract])) OR (Hiatal Hernias, Sliding[Title/Abstract])) OR (Sliding Hiatal Hernias[Title/Abstract])) OR (Sliding Esophageal Hernia[Title/Abstract])) OR (Esophageal Hernia, Sliding[Title/Abstract])) OR (Esophageal Hernias, Sliding[Title/Abstract])) OR (Hernia, Sliding Esophageal[Title/Abstract])) OR (Hernias, Sliding Esophageal[Title/Abstract])) OR (Sliding Esophageal Hernias[Title/Abstract])) OR (Esophageal Hernia[Title/Abstract])) OR (Esophageal Hernias[Title/Abstract])) OR (Hernias, Esophageal[Title/Abstract])) OR (Hernia, Esophageal[Title/Abstract])) OR (Paraesophageal Hiatal Hernia[Title/Abstract])) OR (Hernia, Paraesophageal Hiatal[Title/Abstract])) OR (Hernias, Paraesophageal Hiatal[Title/Abstract])) OR (Hiatal Hernia, Paraesophageal[Title/Abstract])) OR (Hiatal Hernias, Paraesophageal[Title/Abstract])) OR (Paraesophageal Hiatal Hernias[Title/Abstract])) OR (Hernia, Paraesophageal[Title/Abstract])) OR (Hernias, Paraesophageal[Title/Abstract])) OR (Paraesophageal Hernia[Title/Abstract])) OR (Paraesophageal Hernias[Title/Abstract]) | [8,134](https://pubmed.ncbi.nlm.nih.gov/?term=%28%28%28%28%28%28%28%28%28%28%28%28%28%28%28%28%28%28%28%28%28%28%28%28%28%28%28%28%28%28%28%28Hernias%2C+Hiatal%5BTitle%2FAbstract%5D%29+OR+%28Hiatal+Hernias%5BTitle%2FAbstract%5D%29%29+OR+%28Hernia%2C+Hiatus%5BTitle%2FAbstract%5D%29%29+OR+%28Hernias%2C+Hiatus%5BTitle%2FAbstract%5D%29%29+OR+%28Hiatus+Hernia%5BTitle%2FAbstract%5D%29%29+OR+%28Hiatus+Hernias%5BTitle%2FAbstract%5D%29%29+OR+%28Hiatal+Hernia%5BTitle%2FAbstract%5D%29%29+OR+%28Sliding+Hiatal+Hernia%5BTitle%2FAbstract%5D%29%29+OR+%28Hernia%2C+Sliding+Hiatal%5BTitle%2FAbstract%5D%29%29+OR+%28Hernias%2C+Sliding+Hiatal%5BTitle%2FAbstract%5D%29%29+OR+%28Hiatal+Hernia%2C+Sliding%5BTitle%2FAbstract%5D%29%29+OR+%28Hiatal+Hernias%2C+Sliding%5BTitle%2FAbstract%5D%29%29+OR+%28Sliding+Hiatal+Hernias%5BTitle%2FAbstract%5D%29%29+OR+%28Sliding+Esophageal+Hernia%5BTitle%2FAbstract%5D%29%29+OR+%28Esophageal+Hernia%2C+Sliding%5BTitle%2FAbstract%5D%29%29+OR+%28Esophageal+Hernias%2C+Sliding%5BTitle%2FAbstract%5D%29%29+OR+%28Hernia%2C+Sliding+Esophageal%5BTitle%2FAbstract%5D%29%29+OR+%28Hernias%2C+Sliding+Esophageal%5BTitle%2FAbstract%5D%29%29+OR+%28Sliding+Esophageal+Hernias%5BTitle%2FAbstract%5D%29%29+OR+%28Esophageal+Hernia%5BTitle%2FAbstract%5D%29%29+OR+%28Esophageal+Hernias%5BTitle%2FAbstract%5D%29%29+OR+%28Hernias%2C+Esophageal%5BTitle%2FAbstract%5D%29%29+OR+%28Hernia%2C+Esophageal%5BTitle%2FAbstract%5D%29%29+OR+%28Paraesophageal+Hiatal+Hernia%5BTitle%2FAbstract%5D%29%29+OR+%28Hernia%2C+Paraesophageal+Hiatal%5BTitle%2FAbstract%5D%29%29+OR+%28Hernias%2C+Paraesophageal+Hiatal%5BTitle%2FAbstract%5D%29%29+OR+%28Hiatal+Hernia%2C+Paraesophageal%5BTitle%2FAbstract%5D%29%29+OR+%28Hiatal+Hernias%2C+Paraesophageal%5BTitle%2FAbstract%5D%29%29+OR+%28Paraesophageal+Hiatal+Hernias%5BTitle%2FAbstract%5D%29%29+OR+%28Hernia%2C+Paraesophageal%5BTitle%2FAbstract%5D%29%29+OR+%28Hernias%2C+Paraesophageal%5BTitle%2FAbstract%5D%29%29+OR+%28Paraesophageal+Hernia%5BTitle%2FAbstract%5D%29%29+OR+%28Paraesophageal+Hernias%5BTitle%2FAbstract%5D%29&ac=no&sort=relevance) |
| #3 | Search: (((((((((((((((((((((((((((((((((Hernias, Hiatal[Title/Abstract]) OR (Hiatal Hernias[Title/Abstract])) OR (Hernia, Hiatus[Title/Abstract])) OR (Hernias, Hiatus[Title/Abstract])) OR (Hiatus Hernia[Title/Abstract])) OR (Hiatus Hernias[Title/Abstract])) OR (Hiatal Hernia[Title/Abstract])) OR (Sliding Hiatal Hernia[Title/Abstract])) OR (Hernia, Sliding Hiatal[Title/Abstract])) OR (Hernias, Sliding Hiatal[Title/Abstract])) OR (Hiatal Hernia, Sliding[Title/Abstract])) OR (Hiatal Hernias, Sliding[Title/Abstract])) OR (Sliding Hiatal Hernias[Title/Abstract])) OR (Sliding Esophageal Hernia[Title/Abstract])) OR (Esophageal Hernia, Sliding[Title/Abstract])) OR (Esophageal Hernias, Sliding[Title/Abstract])) OR (Hernia, Sliding Esophageal[Title/Abstract])) OR (Hernias, Sliding Esophageal[Title/Abstract])) OR (Sliding Esophageal Hernias[Title/Abstract])) OR (Esophageal Hernia[Title/Abstract])) OR (Esophageal Hernias[Title/Abstract])) OR (Hernias, Esophageal[Title/Abstract])) OR (Hernia, Esophageal[Title/Abstract])) OR (Paraesophageal Hiatal Hernia[Title/Abstract])) OR (Hernia, Paraesophageal Hiatal[Title/Abstract])) OR (Hernias, Paraesophageal Hiatal[Title/Abstract])) OR (Hiatal Hernia, Paraesophageal[Title/Abstract])) OR (Hiatal Hernias, Paraesophageal[Title/Abstract])) OR (Paraesophageal Hiatal Hernias[Title/Abstract])) OR (Hernia, Paraesophageal[Title/Abstract])) OR (Hernias, Paraesophageal[Title/Abstract])) OR (Paraesophageal Hernia[Title/Abstract])) OR (Paraesophageal Hernias[Title/Abstract])) OR ("Hernia, Hiatal"[Mesh]) | [10,356](https://pubmed.ncbi.nlm.nih.gov/?term=%28%28%28%28%28%28%28%28%28%28%28%28%28%28%28%28%28%28%28%28%28%28%28%28%28%28%28%28%28%28%28%28%28Hernias%2C+Hiatal%5BTitle%2FAbstract%5D%29+OR+%28Hiatal+Hernias%5BTitle%2FAbstract%5D%29%29+OR+%28Hernia%2C+Hiatus%5BTitle%2FAbstract%5D%29%29+OR+%28Hernias%2C+Hiatus%5BTitle%2FAbstract%5D%29%29+OR+%28Hiatus+Hernia%5BTitle%2FAbstract%5D%29%29+OR+%28Hiatus+Hernias%5BTitle%2FAbstract%5D%29%29+OR+%28Hiatal+Hernia%5BTitle%2FAbstract%5D%29%29+OR+%28Sliding+Hiatal+Hernia%5BTitle%2FAbstract%5D%29%29+OR+%28Hernia%2C+Sliding+Hiatal%5BTitle%2FAbstract%5D%29%29+OR+%28Hernias%2C+Sliding+Hiatal%5BTitle%2FAbstract%5D%29%29+OR+%28Hiatal+Hernia%2C+Sliding%5BTitle%2FAbstract%5D%29%29+OR+%28Hiatal+Hernias%2C+Sliding%5BTitle%2FAbstract%5D%29%29+OR+%28Sliding+Hiatal+Hernias%5BTitle%2FAbstract%5D%29%29+OR+%28Sliding+Esophageal+Hernia%5BTitle%2FAbstract%5D%29%29+OR+%28Esophageal+Hernia%2C+Sliding%5BTitle%2FAbstract%5D%29%29+OR+%28Esophageal+Hernias%2C+Sliding%5BTitle%2FAbstract%5D%29%29+OR+%28Hernia%2C+Sliding+Esophageal%5BTitle%2FAbstract%5D%29%29+OR+%28Hernias%2C+Sliding+Esophageal%5BTitle%2FAbstract%5D%29%29+OR+%28Sliding+Esophageal+Hernias%5BTitle%2FAbstract%5D%29%29+OR+%28Esophageal+Hernia%5BTitle%2FAbstract%5D%29%29+OR+%28Esophageal+Hernias%5BTitle%2FAbstract%5D%29%29+OR+%28Hernias%2C+Esophageal%5BTitle%2FAbstract%5D%29%29+OR+%28Hernia%2C+Esophageal%5BTitle%2FAbstract%5D%29%29+OR+%28Paraesophageal+Hiatal+Hernia%5BTitle%2FAbstract%5D%29%29+OR+%28Hernia%2C+Paraesophageal+Hiatal%5BTitle%2FAbstract%5D%29%29+OR+%28Hernias%2C+Paraesophageal+Hiatal%5BTitle%2FAbstract%5D%29%29+OR+%28Hiatal+Hernia%2C+Paraesophageal%5BTitle%2FAbstract%5D%29%29+OR+%28Hiatal+Hernias%2C+Paraesophageal%5BTitle%2FAbstract%5D%29%29+OR+%28Paraesophageal+Hiatal+Hernias%5BTitle%2FAbstract%5D%29%29+OR+%28Hernia%2C+Paraesophageal%5BTitle%2FAbstract%5D%29%29+OR+%28Hernias%2C+Paraesophageal%5BTitle%2FAbstract%5D%29%29+OR+%28Paraesophageal+Hernia%5BTitle%2FAbstract%5D%29%29+OR+%28Paraesophageal+Hernias%5BTitle%2FAbstract%5D%29%29+OR+%28%22Hernia%2C+Hiatal%22%5BMesh%5D%252) |
| #4 | Search: "Surgical Procedures, Operative"[Mesh] Sort by: Most Recent | [3,459,251](https://pubmed.ncbi.nlm.nih.gov/?sort=date&term=%22Surgical+Procedures%2C+Operative%22%5BMesh%5D) |
| #5 | Search: (((((((((((((((Operative Procedures[Title/Abstract]) OR (Operative Procedure[Title/Abstract])) OR (Procedure, Operative[Title/Abstract])) OR (Procedures, Operative[Title/Abstract])) OR (Surgical Procedure, Operative[Title/Abstract])) OR (Operative Surgical Procedures[Title/Abstract])) OR (Procedure, Operative Surgical[Title/Abstract])) OR (Procedures, Operative Surgical[Title/Abstract])) OR (Surgical Procedures[Title/Abstract])) OR (Procedure, Surgical[Title/Abstract])) OR (Procedures, Surgical[Title/Abstract])) OR (Surgical Procedure[Title/Abstract])) OR (Operative Surgical Procedure[Title/Abstract])) OR (Surgery, Ghost[Title/Abstract])) OR (Ghost Surgery[Title/Abstract])) OR ("Surgical Procedures, Operative"[Mesh]) | 127,038 |
| #6 | Search: (((((((((((((((Operative Procedures[Title/Abstract]) OR (Operative Procedure[Title/Abstract])) OR (Procedure, Operative[Title/Abstract])) OR (Procedures, Operative[Title/Abstract])) OR (Surgical Procedure, Operative[Title/Abstract])) OR (Operative Surgical Procedures[Title/Abstract])) OR (Procedure, Operative Surgical[Title/Abstract])) OR (Procedures, Operative Surgical[Title/Abstract])) OR (Surgical Procedures[Title/Abstract])) OR (Procedure, Surgical[Title/Abstract])) OR (Procedures, Surgical[Title/Abstract])) OR (Surgical Procedure[Title/Abstract])) OR (Operative Surgical Procedure[Title/Abstract])) OR (Surgery, Ghost[Title/Abstract])) OR (Ghost Surgery[Title/Abstract])) OR ("Surgical Procedures, Operative"[Mesh]) | 3,509,377 |
| #7 | Search: (((((symptom improvement[Title/Abstract]) OR (outcome*[Title/Abstract])) OR (complication*[Title/Abstract])) OR (quality of life[Title/Abstract])) OR (pulmonary[Title/Abstract])) OR (dyspnea[Title/Abstract]) | 3,735,202 |
| #8 | Search: (((((((symptom improvement[Title/Abstract]) OR (outcome*[Title/Abstract])) OR (complication*[Title/Abstract])) OR (quality of life[Title/Abstract])) OR (pulmonary[Title/Abstract])) OR (dyspnea[Title/Abstract])) AND ((((((((((((((((Operative Procedures[Title/Abstract]) OR (Operative Procedure[Title/Abstract])) OR (Procedure, Operative[Title/Abstract])) OR (Procedures, Operative[Title/Abstract])) OR (Surgical Procedure, Operative[Title/Abstract])) OR (Operative Surgical Procedures[Title/Abstract])) OR (Procedure, Operative Surgical[Title/Abstract])) OR (Procedures, Operative Surgical[Title/Abstract])) OR (Surgical Procedures[Title/Abstract])) OR (Procedure, Surgical[Title/Abstract])) OR (Procedures, Surgical[Title/Abstract])) OR (Surgical Procedure[Title/Abstract])) OR (Operative Surgical Procedure[Title/Abstract])) OR (Surgery, Ghost[Title/Abstract])) OR (Ghost Surgery[Title/Abstract])) OR ("Surgical Procedures, Operative"[Mesh]))) AND ((((((((((((((((((((((((((((((((((Hernias, Hiatal[Title/Abstract]) OR (Hiatal Hernias[Title/Abstract])) OR (Hernia, Hiatus[Title/Abstract])) OR (Hernias, Hiatus[Title/Abstract])) OR (Hiatus Hernia[Title/Abstract])) OR (Hiatus Hernias[Title/Abstract])) OR (Hiatal Hernia[Title/Abstract])) OR (Sliding Hiatal Hernia[Title/Abstract])) OR (Hernia, Sliding Hiatal[Title/Abstract])) OR (Hernias, Sliding Hiatal[Title/Abstract])) OR (Hiatal Hernia, Sliding[Title/Abstract])) OR (Hiatal Hernias, Sliding[Title/Abstract])) OR (Sliding Hiatal Hernias[Title/Abstract])) OR (Sliding Esophageal Hernia[Title/Abstract])) OR (Esophageal Hernia, Sliding[Title/Abstract])) OR (Esophageal Hernias, Sliding[Title/Abstract])) OR (Hernia, Sliding Esophageal[Title/Abstract])) OR (Hernias, Sliding Esophageal[Title/Abstract])) OR (Sliding Esophageal Hernias[Title/Abstract])) OR (Esophageal Hernia[Title/Abstract])) OR (Esophageal Hernias[Title/Abstract])) OR (Hernias, Esophageal[Title/Abstract])) OR (Hernia, Esophageal[Title/Abstract])) OR (Paraesophageal Hiatal Hernia[Title/Abstract])) OR (Hernia, Paraesophageal Hiatal[Title/Abstract])) OR (Hernias, Paraesophageal Hiatal[Title/Abstract])) OR (Hiatal Hernia, Paraesophageal[Title/Abstract])) OR (Hiatal Hernias, Paraesophageal[Title/Abstract])) OR (Paraesophageal Hiatal Hernias[Title/Abstract])) OR (Hernia, Paraesophageal[Title/Abstract])) OR (Hernias, Paraesophageal[Title/Abstract])) OR (Paraesophageal Hernia[Title/Abstract])) OR (Paraesophageal Hernias[Title/Abstract])) OR ("Hernia, Hiatal"[Mesh])) | 1,809 |
| #9 | Search: (((((((symptom improvement[Title/Abstract]) OR (outcome*[Title/Abstract])) OR (complication*[Title/Abstract])) OR (quality of life[Title/Abstract])) OR (pulmonary[Title/Abstract])) OR (dyspnea[Title/Abstract])) AND ((((((((((((((((Operative Procedures[Title/Abstract]) OR (Operative Procedure[Title/Abstract])) OR (Procedure, Operative[Title/Abstract])) OR (Procedures, Operative[Title/Abstract])) OR (Surgical Procedure, Operative[Title/Abstract])) OR (Operative Surgical Procedures[Title/Abstract])) OR (Procedure, Operative Surgical[Title/Abstract])) OR (Procedures, Operative Surgical[Title/Abstract])) OR (Surgical Procedures[Title/Abstract])) OR (Procedure, Surgical[Title/Abstract])) OR (Procedures, Surgical[Title/Abstract])) OR (Surgical Procedure[Title/Abstract])) OR (Operative Surgical Procedure[Title/Abstract])) OR (Surgery, Ghost[Title/Abstract])) OR (Ghost Surgery[Title/Abstract])) OR ("Surgical Procedures, Operative"[Mesh]))) AND ((((((((((((((((((((((((((((((((((Hernias, Hiatal[Title/Abstract]) OR (Hiatal Hernias[Title/Abstract])) OR (Hernia, Hiatus[Title/Abstract])) OR (Hernias, Hiatus[Title/Abstract])) OR (Hiatus Hernia[Title/Abstract])) OR (Hiatus Hernias[Title/Abstract])) OR (Hiatal Hernia[Title/Abstract])) OR (Sliding Hiatal Hernia[Title/Abstract])) OR (Hernia, Sliding Hiatal[Title/Abstract])) OR (Hernias, Sliding Hiatal[Title/Abstract])) OR (Hiatal Hernia, Sliding[Title/Abstract])) OR (Hiatal Hernias, Sliding[Title/Abstract])) OR (Sliding Hiatal Hernias[Title/Abstract])) OR (Sliding Esophageal Hernia[Title/Abstract])) OR (Esophageal Hernia, Sliding[Title/Abstract])) OR (Esophageal Hernias, Sliding[Title/Abstract])) OR (Hernia, Sliding Esophageal[Title/Abstract])) OR (Hernias, Sliding Esophageal[Title/Abstract])) OR (Sliding Esophageal Hernias[Title/Abstract])) OR (Esophageal Hernia[Title/Abstract])) OR (Esophageal Hernias[Title/Abstract])) OR (Hernias, Esophageal[Title/Abstract])) OR (Hernia, Esophageal[Title/Abstract])) OR (Paraesophageal Hiatal Hernia[Title/Abstract])) OR (Hernia, Paraesophageal Hiatal[Title/Abstract])) OR (Hernias, Paraesophageal Hiatal[Title/Abstract])) OR (Hiatal Hernia, Paraesophageal[Title/Abstract])) OR (Hiatal Hernias, Paraesophageal[Title/Abstract])) OR (Paraesophageal Hiatal Hernias[Title/Abstract])) OR (Hernia, Paraesophageal[Title/Abstract])) OR (Hernias, Paraesophageal[Title/Abstract])) OR (Paraesophageal Hernia[Title/Abstract])) OR (Paraesophageal Hernias[Title/Abstract])) OR ("Hernia, Hiatal"[Mesh])) Filters: from 2000 - 2022 | 1,485 |

**Table 2** Cochrane

| No. | Content | result |
| --- | --- | --- |
| #1 | MeSH descriptor: [Hernia, Hiatal] explode all trees | 98 |
| #2 | (Hernias, Hiatal OR Hiatal Hernias OR Hernia, Hiatus OR Hernias, Hiatus OR Hiatus Hernia OR Hiatus Hernias OR Hiatal Hernia OR Sliding Hiatal Hernia OR Hernia, Sliding Hiatal OR Hernias, Sliding Hiatal OR Hiatal Hernia, Sliding OR Hiatal Hernias, Sliding OR Sliding Hiatal Hernias OR Sliding Esophageal Hernia OR Esophageal Hernia, Sliding OR Esophageal Hernias, Sliding OR Hernia, Sliding Esophageal OR Hernias, Sliding Esophageal OR Sliding Esophageal Hernias OR Esophageal Hernia OR Esophageal Hernias OR Hernias, Esophageal OR Hernia, Esophageal OR Paraesophageal Hiatal Hernia OR Hernia, Paraesophageal Hiatal OR Hernias, Paraesophageal Hiatal OR Hiatal Hernia, Paraesophageal OR Hiatal Hernias, Paraesophageal OR Paraesophageal Hiatal Hernias OR Hernia, Paraesophageal OR Hernias, Paraesophageal OR Paraesophageal Hernia OR Paraesophageal Hernias):ti,ab,kw | 465 |
| #3 | #1 OR #2 | 465 |
| #4 | MeSH descriptor: [Surgical Procedures, Operative] explode all trees | 129624 |
| #5 | (Operative Procedures OR Operative Procedure OR Procedure, Operative OR Procedures, Operative OR Surgical Procedure, Operative OR Operative Surgical Procedures OR Procedure, Operative Surgical OR Procedures, Operative Surgical OR Surgical Procedures OR Procedure, Surgical OR Procedures, Surgical OR Surgical Procedure OR Operative Surgical Procedure OR Surgery, Ghost OR Ghost Surgery):ti,ab,kw | 66289 |
| #6 | #4 OR #5 | 169395 |
| #7 | (Symptom improvement OR Outcomes OR Complications OR Quality of life OR Pulmonary OR dyspnea):ti,ab,kw | 896016 |
| #8 | #3 AND #6 AND #7 | 169 |

**Table 3** Embase

| No. | Content | Result |
| --- | --- | --- |
| #1 | 'hiatus hernia'/exp | 14,490 |
| #2 | 'hernias, hiatal' OR 'hiatal hernias' OR 'hernia, hiatus'/exp OR 'hernia, hiatus' OR 'hernias, hiatus' OR 'hiatus hernia'/exp OR 'hiatus hernia' OR 'hiatus hernias' OR 'hiatal hernia'/exp OR 'hiatal hernia' OR 'sliding hiatal hernia' OR 'hernia, sliding hiatal' OR 'hernias, sliding hiatal' OR 'hiatal hernia, sliding' OR 'hiatal hernias, sliding' OR 'sliding hiatal hernias' OR 'sliding esophageal hernia' OR 'esophageal hernia, sliding' OR 'esophageal hernias, sliding' OR 'hernia, sliding esophageal' OR 'hernias, sliding esophageal' OR 'sliding esophageal hernias' OR 'esophageal hernia' OR 'esophageal hernias' OR 'hernias, esophageal' OR 'hernia, esophageal' OR 'paraesophageal hiatal hernia'/exp OR 'paraesophageal hiatal hernia' OR 'hernia, paraesophageal hiatal' OR 'hernias, paraesophageal hiatal' OR 'hiatal hernia, paraesophageal' OR 'hiatal hernias, paraesophageal' OR 'paraesophageal hiatal hernias' OR 'hernia, paraesophageal' OR 'hernias, paraesophageal' OR 'paraesophageal hernia'/exp OR 'paraesophageal hernia' OR 'paraesophageal hernias':ti,ab | 17,555 |
| #3 | #1 OR #2 | 17,555 |
| #4 | 'surgery'/exp | 5,797,200 |
| #5 | 'operative procedures' OR 'operative procedure' OR 'procedure, operative' OR 'procedures, operative' OR 'surgical procedure, operative' OR 'operative surgical procedures'/exp OR 'operative surgical procedures' OR 'procedure, operative surgical' OR 'procedures, operative surgical' OR 'surgical procedures' OR 'procedure, surgical' OR 'procedures, surgical' OR 'surgical procedure'/exp OR 'surgical procedure' OR 'operative surgical procedure'/exp OR 'operative surgical procedure' OR 'surgery, ghost' OR 'ghost surgery':ti,ab | 5,826,140 |
| #6 | #4 OR #5 | 5,826,140 |
| #7 | 'symptom improvement' OR 'outcomes' OR 'complications' OR 'quality of life' OR 'pulmonary' OR 'dyspnea':ti,ab | 4,518,662 |
| #8 | #3 AND #6 AND #7 | 4,418 |

**Table 4** Web of Science

| #1 | ((TS=(Hernia, Hiatal OR Hernias, Hiatal OR Hiatal Hernias OR Hernia, Hiatus OR Hernias, Hiatus OR Hiatus Hernia OR Hiatus Hernias OR Hiatal Hernia OR Sliding Hiatal Hernia OR Hernia, Sliding Hiatal OR Hernias, Sliding Hiatal OR Hiatal Hernia, Sliding OR Hiatal Hernias, Sliding OR Sliding Hiatal Hernias OR Sliding Esophageal Hernia OR Esophageal Hernia, Sliding OR Esophageal Hernias, Sliding OR Hernia, Sliding Esophageal OR Hernias, Sliding Esophageal OR Sliding Esophageal Hernias OR Esophageal Hernia OR Esophageal Hernias OR Hernias, Esophageal OR Hernia, Esophageal OR Paraesophageal Hiatal Hernia OR Hernia, Paraesophageal Hiatal OR Hernias, Paraesophageal Hiatal OR Hiatal Hernia, Paraesophageal OR Hiatal Hernias, Paraesophageal OR Paraesophageal Hiatal Hernias OR Hernia, Paraesophageal OR Hernias, Paraesophageal OR Paraesophageal Hernia OR Paraesophageal Hernias)) AND TS=(Surgical Procedures, Operative OR Operative Procedures OR Operative Procedure OR Procedure, Operative OR Procedures, Operative OR Surgical Procedure, Operative OR Operative Surgical Procedures OR Procedure, Operative Surgical OR Procedures, Operative Surgical OR Surgical Procedures OR Procedure, Surgical OR Procedures, Surgical OR Surgical Procedure OR Operative Surgical Procedure OR Surgery, Ghost OR Ghost Surgery)) AND TS=(symptom improvement OR outcomes OR complications OR quality of life OR pulmonary OR dyspnea) | 632 |
| --- | --- | --- |

**Fig. 1** the results of sensitivity analysis for FEV1


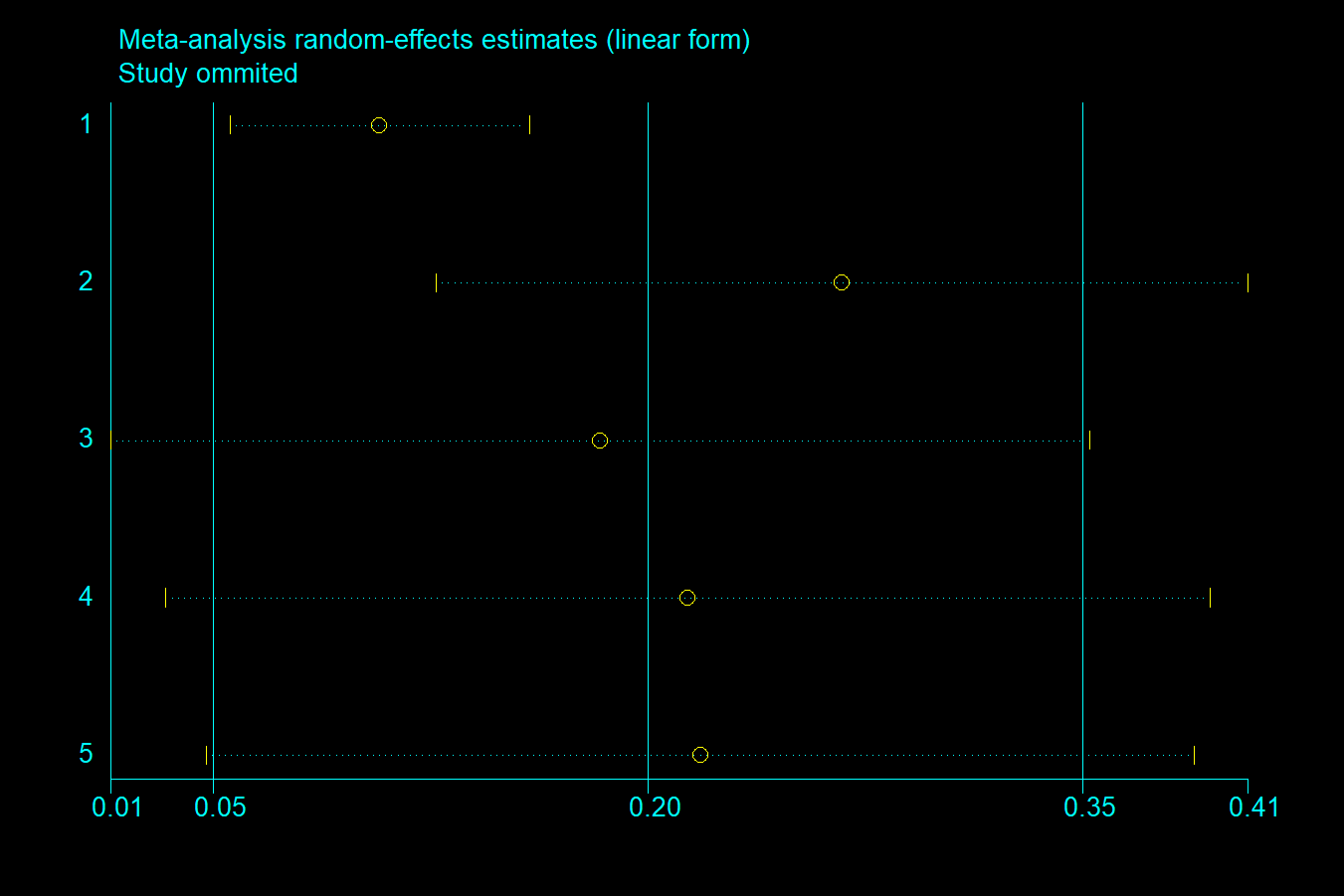


1: Aiolfi, A. 2022

2: Bouriez, D. 2022

3: Low, D. E. 2002

4: Naoum, C. 2017

5: Naoum, C. 2011

**Fig. 2** the results of Egger’ s test


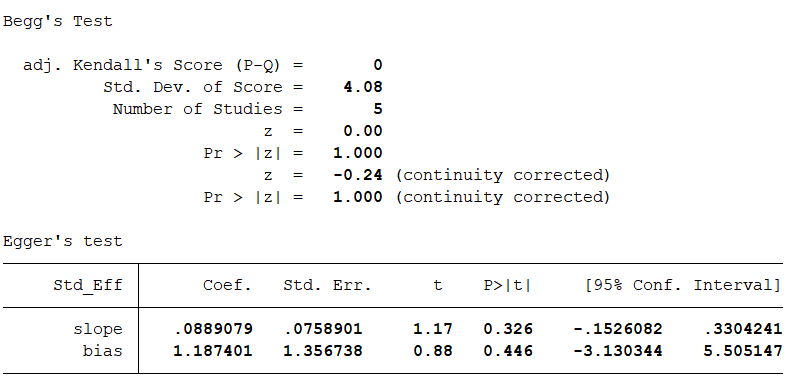

Supplement: Supplementary file 1 — Supplementary file1 (DOCX 49 KB) [file 10029_2023_2756_MOESM1_ESM.docx]
